# Higher Viral Stability and Ethanol Resistance of Avian Influenza A(H5N1) Virus on Human Skin

**DOI:** 10.3201/eid2803.211752

**Published:** 2022-03

**Authors:** Risa Bandou, Ryohei Hirose, Takaaki Nakaya, Hajime Miyazaki, Naoto Watanabe, Takuma Yoshida, Tomo Daidoji, Yoshito Itoh, Hiroshi Ikegaya

**Affiliations:** Graduate School of Medical Science, Kyoto Prefectural University of Medicine, Kyoto, Japan

**Keywords:** H5N1, avian influenza virus, human skin, stability, disinfectant effectiveness, hand hygiene, influenza, viruses, zoonoses, Japan, antimicrobial resistance, avian influenza A(H5N1)

## Abstract

Evaluating the stability of highly pathogenic avian influenza viruses on human skin and measuring the effectiveness of disinfectants are crucial for preventing contact disease transmission. We constructed an evaluation model using autopsy skin samples and evaluated factors that affect the stability and disinfectant effectiveness for various subtypes. The survival time of the avian influenza A(H5N1) virus on plastic surfaces was ≈26 hours and on skin surfaces ≈4.5 hours, >2.5-fold longer than other subtypes. The effectiveness of a relatively low ethanol concentration (32%–36% wt/wt) against the H5N1 subtype was substantially reduced compared with other subtypes. Moreover, recombinant viruses with the neuraminidase gene of H5N1 survived longer on plastic and skin surfaces than other recombinant viruses and were resistant to ethanol. Our results imply that the H5N1 subtype poses a higher contact transmission risk because of its higher stability and ethanol resistance, which might depend on the neuraminidase protein.

Highly pathogenic subtypes of avian influenza virus (AIV) can infect humans and cause fatal respiratory failure (*1*–*3*). Since 2003, cases of avian influenza A(H5N1) and avian influenza A(H7N9) transmission from birds to humans have been confirmed in the Middle East, West Africa, Europe, and Asia. In >50% of these cases, the outcome was fatal (*4*,*5*). Recently, subtype H5N6, H5N8, and H9N2 AIVs have been confirmed to infect humans (*6*,*7*). The H5N9 subtype has also been reported to be highly transmissible (*8*). Most of these cases of AIV infection have been caused by contact transmission from infected birds (*9*–*14*). Therefore, preventing contact transmission is crucial for controlling the spread of AIV infection.

Knowledge of viral stability is vital to understanding contact transmission (*15*,*16*), and several studies have assessed the stability of AIVs under various conditions (*17*–*25*). Viral stability has been reported to decrease under conditions of high temperature, high salinity, or low pH (*17*,*19*,*21*–*25*). However, because contact transmission occurs when the virus enters the human body through the skin, evaluating the stability, or survival time, of AIV on human skin and the effectiveness of disinfectants against AIV on skin are essential to assess contact-transmission risks and develop more effective infection control methods (*26*–*29*). However, clinical research in this regard is limited because of the risks involved in applying highly pathogenic AIV directly to the skin of human study participants. Therefore, the stability of AIVs and the efficacy of related disinfectants remain unknown.

Moreover, although previous studies have suggested that the stability of different AIV subtypes might vary, these differences were not clearly defined (*20*–*22*,*25*). Current contact transmission control methods are based on the assumption that no great differences in stability among AIV subtypes or in the effectiveness of available disinfectants against them exist (*30*,*31*). If substantial differences exist in terms of stability and disinfectant effectiveness among subtypes, then the optimal infection control methods might differ for each subtype. Therefore, developing optimal methods for controlling the transmission of each subtype requires an accurate analysis of the differences among subtypes.

An ex vivo evaluation model using skin collected from autopsy specimens has been developed that accurately and safely assesses the stability of highly pathogenic pathogens and the effectiveness of different disinfectants (*26*–*28*). In this study, we aimed to elucidate the differences in the stability of AIV subtypes and disinfectant efficacy against AIV on the surface of human skin by using this constructed model. Furthermore, we aimed to elucidate the genetic mechanisms responsible for stability differences among subtypes by using recombinant viruses.

## Methods

### Viruses

Recombinant H5N1 viruses with the neuraminidase (NA) or hemagglutinin (HA) gene of the H5N3 subtypes (rH5N1-H5N3-NA and rH5N1-H5N3-HA), or recombinant H5N3 viruses with the NA, HA, nonstructural protein (NS), or matrix protein (M) gene of the H5N1 subtypes (rH5N3-H5N1-NA, rH5N3-H5N1-NS, rH5N3-H5N1-M, and rH5N3-H5N1-HA) were generated as target viruses by using a reverse-genetics system. We evaluated the recombinant viruses A/crow/Kyoto/53/04(H5N1) (H5N1-Ky), A/chicken/Egypt/CL6/07(H5N1) (H5N1-Eg), A/Anhui/1/23(H7N9) (H7N9), A/duck/Hong Kong/820/80(H5N3) (H5N3), A/turkey/Ontario/7732/66(H5N9) (H5N9), a clinical H3N2 strain (H3N2), A/Puerto Rico/8/1934(H1N1) (H1N1-PR8), and A/Osaka/64/2009 (H1N1-Ok-pdm).

### Constructing a Model to Evaluate Virus Stability and Disinfectant Effectiveness

Human skin was collected from forensic autopsy specimens obtained from the Department of Forensic Medicine, Kyoto Prefectural University of Medicine (Kyoto, Japan). Abdominal skin specimens from subjects from 20–70 years of age were cut into squares with approximate dimensions of 4 cm × 8 cm. Autopsy specimens in which the skin was considerably damaged by burning or drowning were excluded (*26*,*32*). Collected skin can reportedly be used for grafting even 24 hours after death, and within 36 hours of excision, the skin retains its physiologic function relatively well with no change in cell viability after 14 days in culture (*33*–*35*). Therefore, in this study, skin specimens were obtained at ≈1 day after death to preserve the physiologic function of the epidermis. By using the skin autopsy specimens, we developed an ex vivo model to evaluate the stability of different viruses on the surface of human skin and the effectiveness of different disinfectants against viruses on skin. Skin from which the panniculus adiposus had been removed was washed with phosphate-buffered saline (PBS) and placed in a culture insert (Corning Inc., https://www.corning.com) on a membrane with a pore size of 8.0 µm. The culture inserts were placed in six-well plates containing 1.0 mL of Dulbecco modified Eagle’s medium (DMEM) (Sigma-Aldrich, https://www.sigmaaldrich.com) (*26*,*27*).

### Evaluation of Viral Stability

We evaluated virus survival on plastic and human skin surfaces. Virus solutions (2.0 × 10^5^ focus-forming units [FFUs] in 2 µL of PBS) were applied to the surface of plastic or human skin (the constructed evaluation model). Each sample was incubated in a controlled environment (25°C and 45%–55% relative humidity) for 0–24 h. The virus remaining on the surface was then collected in 1.0 mL of DMEM and titrated (*15*,*26*,*28*,*36*,*37*). The detection limit for the titer of the virus remaining on the surface was 10^1^ FFUs. For each condition, we performed 3 independent experiments, and the titer values are expressed as mean + SD of the mean. The elapsed time was used as the explanatory variable (x-axis) and the logarithmic virus titer was used as the explained variable (y-axis). Least-squares linear-regression analysis was performed by using a logarithmic link function to create regression curves for both viruses. Because the detection limit of each influenza virus titer was 10^1^ FFUs, the X value (when the Y value of the regression curve was 1.0) was used as the survival time. The half-life of each virus was calculated from the slope of each regression curve when the amount of virus remaining on the surface was 2, 3, or 4 log_10_ FFUs (*26*,*28*).

### Evaluation of Disinfectant Effectiveness

We evaluated the effectiveness of available disinfectants against influenza viruses. The disinfectants evaluated were 20%, 32%, 34%, 36%, 40%, 60%, and 80% (wt/wt) ethyl alcohol (EA); 70% (wt/wt) isopropanol (IPA); 0.05% and 0.2% (wt/vol) benzalkonium chloride (BAC); and 0.2% and 1.0% (wt/vol) chlorhexidine gluconate (CHG).

In a 1.5-mL tube, we mixed 5 µL of PBS containing either avian or human influenza virus (4.0 × 10^5^ FFUs) with 95 µL of various disinfectants for 15 or 60 s. Subsequently, we neutralized the resulting solutions with 900 µL of Soybean–Casein Digest Broth prepared with Lecithin and Polysorbate 80 (SCLDP) medium. Thereafter, we added 3 mL of DMEM to the neutralized solution and measured the remaining viral titers (*27*,*38*–*40*). The detection limit for the virus titers was 10^1.6^ FFUs.

We used the same disinfectants for in vitro evaluations and ex vivo evaluations. We applied each virus solution (2 µL of PBS containing 2.0 × 10^5^ FFUs of virus) to the skin specimens (the constructed evaluation model), then incubated each skin sample for 15 min at 25°C under 45%–55% relative humidity to completely dry the viral mixture on the skin. Subsequently, we immersed each skin sample surface in 1 mL of the disinfectant for 15 or 60 s and then air-dried for 5 min. After drying, we recovered the remaining viruses on the skin with 250 µL of SCDLP and 750 µL of DMEM and measured the remaining viral load (*26*,*27*). The detection limit for the virus titers was 10^1^ FFUs.

To determine the effectiveness of the disinfectants under each condition, we calculated logarithmic reductions of the virus titers with normalization to the PBS control. We performed 3 independent experiments for each condition, and the results are expressed as mean + SD of the mean (Appendix). The research protocol, including the sampling method, was reviewed and approved by the Institutional Review Board of Kyoto Prefectural University of Medicine (approval no. ERB-C-1593).

## Results

### Stability of Influenza Virus on Plastic

**Figure 1:**
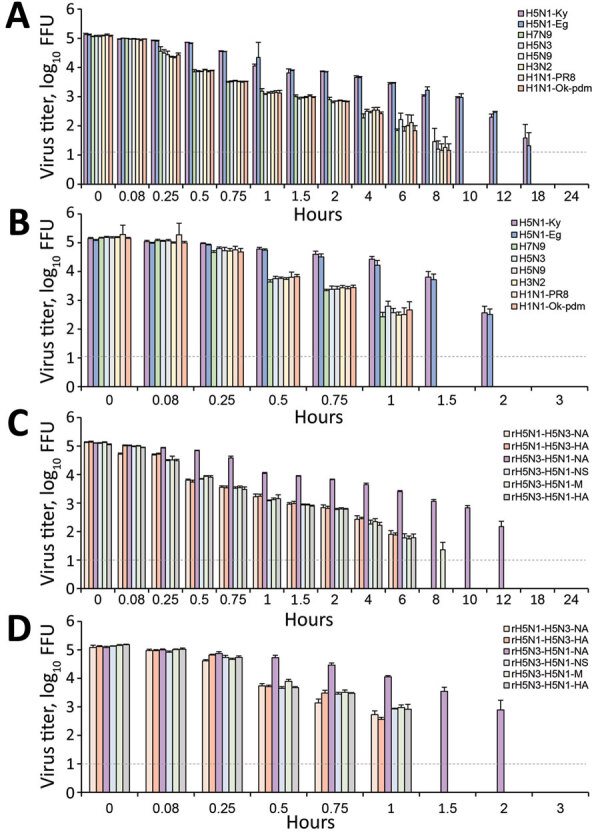
Decrease in titers of influenza virus on plastic (A, C) and the human skin (B, D) surfaces as a function of time. Various subtypes of influenza viruses (A, B) and recombinant viruses (C, D) were targeted. Each virus (2.0 × 10^5^ FFUs) was mixed with 2 µL of phosphate-buffered saline and applied on each surface. Each surface was incubated in a controlled environment (temperature 25°C, humidity 45%–55%) for 0–24 h. The virus on the surface was then recovered in 1 mL of medium and titrated to calculate the titer of virus remaining on the surface. For each condition, 3 independent experiments were performed; results are expressed as mean + SD of the mean. Dotted horizontal lines represent detection limit titers; data below this limit were omitted. Data are shown for H5N1-Ky, A/crow/Kyoto/53/04 (H5N1); H5N1-Eg, A/chicken/Egypt/CL6/07 (H5N1); H7N9, A/Anhui/1/23 (H7N9); H5N3, A/duck/Hong Kong/820/80 (H5N3); H5N9, A/turkey/Ontario/7732/66 (H5N9); H3N2, a clinical strain (H3N2); H1N1-PR8, A/Puerto Rico/8/1934 (H1N1); and H1N1-Ok-pdm, A/Osaka/64/2009 (H1N1). A/crow/Kyoto/53/04 (H5N1) was recombined with the neuraminidase or hemagglutinin gene of AdDuck/Hong Kong/820/80 (H5N3), and the recombinant viruses were designated as rH5N1-H5N3-NA or rH5N1-H5N3-HA, respectively. In addition, A/Duck/Hong Kong/820/80 (H5N3) was recombined with the neuraminidase, nonstructural protein, matrix protein, or hemagglutinin gene of A/crow/Kyoto/53/04 (H5N1), and the recombinant viruses were designated as rH5N3-H5N1-NA, rH5N3-H5N1-NS, rH5N3-H5N1-M, or rH5N3-H5N1-HA. FFU, focus-forming unit.

All influenza virus subtypes except for H5N1 were completely inactive within 10 hours. In contrast, the H5N1 subtype strains tested (H5N1-Ky and H5N1-Eg) remained infectious on the plastic surface after 10 hours but were completely inactive within 24 hours. In addition, the titers of H5N1-Ky and H5N1-Eg remaining on the plastic surface were significantly higher than those of other subtypes at most time points (Figure 1, panel A).

**Figure 2:**
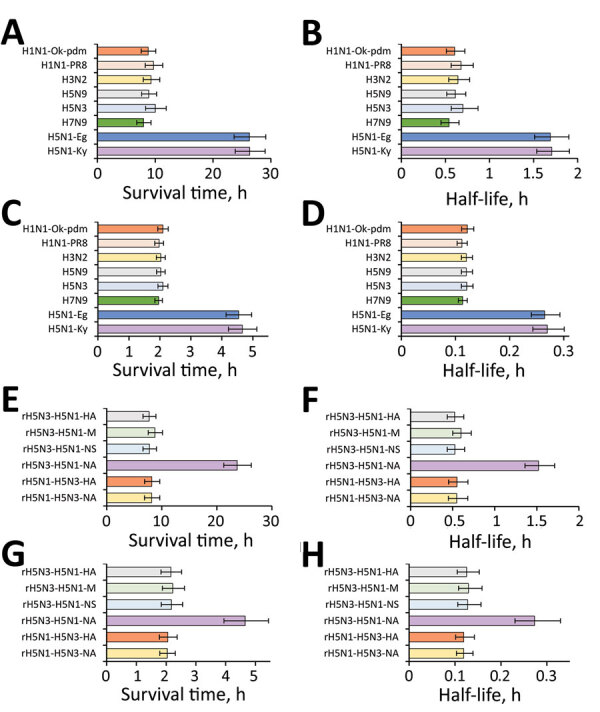
Survival times and half-lives of influenza viruses on plastic and human skin. A, B) Survival times (A) and half-lives (B) of various subtypes of influenza viruses on a plastic surface (Table 1). C, D) Survival times (C) and half-lives (D) of various subtypes of influenza viruses on the surface of human skin (Table 2). E, F) Survival times (E) and half-lives (F) of various recombinant viruses on plastic surfaces (Table 3). G, H) Survival times (G) and half-lives H) of various recombinant viruses on the surface of human skin (Table 4). Survival time is defined as the time until virus on the surface is no longer detected. All half-lives in the graphs refer to the half-life when 10^3^ focus-forming units of virus remained on the skin surface. Data are expressed as median + 95% CI. Data are presented for H5N1-Ky, A/crow/Kyoto/53/04 (H5N1); H5N1-Eg, A/chicken/Egypt/CL6/07 (H5N1); H7N9, A/Anhui/1/23 (H7N9); H5N3, A/duck/Hong Kong/820/80 (H5N3); H5N9, A/turkey/Ontario/7732/66 (H5N9); H3N2, a clinical strain (H3N2); H1N1-PR8, A/Puerto Rico/8/1934 (H1N1); and H1N1-Ok-pdm, A/Osaka/64/2009 (H1N1). A/crow/Kyoto/53/04 (H5N1) was recombined with the neuraminidase or hemagglutinin gene of A/Duck/Hong Kong/820/80 (H5N3), and the recombinant viruses were designated as rH5N1-H5N3-NA or rH5N1-H5N3-HA. In addition, A/Duck/Hong Kong/820/80 (H5N3) was recombined with the neuraminidase, nonstructural protein, matrix protein, or hemagglutinin gene of A/crow/Kyoto/53/04 (H5N1), and the recombinant viruses were designated as rH5N3-H5N1-NA, rH5N3-H5N1-NS, rH5N3-H5N1-M, or rH5N3-H5N1-HA.

Next, we calculated the survival times and half-lives of the virus titers for the virus samples remaining on the surface. The survival times of all subtypes (except for the H5N1 subtype) were ≈8–10 hours. For example, the survival time of the H5N3 subtype was 10.01 (95% CI 8.35–11.91) hours. In contrast, the survival time of H5N1-Ky was 26.35 (95% CI 23.84–29.01) hours and survival time of H5N1-Eg was 26.30 (95% CI 23.64–29.14) hours, both significantly longer than those for other subtypes (Table 1; Figure 2, panel A). Moreover, the half-lives of the H5N1-Ky and H5N1-Eg strains were more than twice as long as those of other subtypes (Table 1; Figure 2, panel B).

**Table 1:** Survival times and half-lives of various subtypes of influenza viruses on a plastic surface*

Subtype	Median survival time (95% CI), h†	Median half-life (95% CI), h‡		
4 (log_10_ FFU)	3 (log_10_ FFU)	2 (log_10_ FFU)
H5N1-Ky	26.35 (23.84–29.01)	1.28 (1.15–1.43)	1.71 (1.54–1.91)	2.56 (2.30–2.86)
H5N1-Eg	26.30 (23.64–29.14)	1.27 (1.13–1.43)	1.69 (1.51–1.90)	2.54 (2.27–2.85)
H7N9	7.97 (6.82–9.27)	0.40 (0.34–0.49)	0.54 (0.45–0.65)	0.81 (0.67–0.98)
H5N3	10.01 (8.35–11.91)	0.52 (0.42–0.65)	0.70 (0.57–0.87)	1.05 (0.85–1.30)
H5N9	8.88 (7.67–10.23)	0.46 (0.39–0.55)	0.61 (0.51–0.73)	0.92 (0.77–1.09)
H3N2	9.28 (7.94–10.79)	0.48 (0.40–0.58)	0.64 (0.54–0.77)	0.96 (0.80–1.16)
H1N1-PR8	9.70 (8.29–11.30)	0.51 (0.42–0.61)	0.68 (0.56–0.82)	1.01 (0.85–1.22)
H1N1-Ok-pdm	8.78 (7.60–10.10)	0.45 (0.38–0.54)	0.60 (0.51–0.72)	0.91 (0.76–1.08)

*FFU, focus-forming units; H5N1-Ky, A/crow/Kyoto/53/04 (H5N1); H5N1-Eg, A/chicken/Egypt/CL6/07 (H5N1); H7N9, A/Anhui/1/23 (H7N9); H5N3, A/Duck/Hong Kong/820/80 (H5N3); H5N9, A/Turkey/Ontario/7732/66 (H5N9); H3N2, Clinical strain (H3N2); H1N1-PR8, A/Puerto Rico /8/1934 (H1N1); H1N1-Ok-pdm, A/Osaka/64/2009 (H1N1). †The elapsed time was used as the explanatory variable (x-axis), and the logarithmic virus titer was used as the explained variable (y-axis). A linear regression analysis with logarithmic link function was performed for each virus to create a curve of regression (Appendix Figure 1). Because the detection limit of each influenza virus titer was 10^1^ FFUs, the X value (when the Y value of the regression curve was 1.0) was used as the survival times. ‡The half-life of each virus was calculated from the slope of each regression curve when the amount of virus remaining on the surface was 2, 3, or 4 log_10_ FFUs.

### Stability of Influenza Virus on Human Skin Surface

All subtypes (except H5N1) were completely inactive within 1.5 hours. In contrast, the H5N1-Ky and H5N1-Eg stains remained active on the skin even after 1.5 hours but were completely inactive within 3 hours. In addition, the titers of H5N1-Ky and H5N1-Eg remaining on the skin were substantially higher than those of other subtypes (Figure 1, panel B).

The survival times of all subtypes (except H5N1) were ≈2 hours. For example, the survival time of the H5N3 subtype was 2.10 (95% CI 1.94–2.26) hours. In contrast, the survival time of H5N1-Ky was 4.66 (95% CI 4.21–5.13) hours and survival time of H5N1-Eg was 4.54 (95% CI 4.16–4.97) hours, both of which were significantly longer than those of the other subtypes studied (Table 2; Figure 2, panel C). Furthermore, the half-life showed the same tendency as the survival time, and the half-lives of H5N1-Ky and H5N1-Eg were more than twice as long as those of other subtypes (Table 2; Figure 2, panel D).

**Table 2:** Survival times and half-lives of various subtypes of influenza viruses on the surface of human skin*

Subtype	Median survival time (95% CI), h†	Median half-life (95% CI), h‡		
4 (log_10_ FFU)	3 (log_10_ FFU)	2 (log_10_ FFU)
H5N1-Ky	4.66 (4.21–5.13)	0.20 (0.18–0.23)	0.27 (0.24–0.30)	0.40 (0.36–0.45)
H5N1-Eg	4.54 (4.14–4.97)	0.20 (0.18–0.22)	0.26 (0.24–0.29)	0.40 (0.36–0.44)
H7N9	1.96 (1.84–2.08)	0.08 (0.08–0.09)	0.11 (0.11–0.12)	0.17 (0.16–0.18)
H5N3	2.10 (1.94–2.26)	0.09 (0.08–0.10)	0.12 (0.11–0.13)	0.18 (0.17–0.20)
H5N9	2.03 (1.89–2.17)	0.09 (0.08–0.09)	0.12 (0.11–0.13)	0.18 (0.16–0.19)
H3N2	2.03 (1.89–2.17)	0.09 (0.08–0.10)	0.12 (0.11–0.13)	0.18 (0.16–0.19)
H1N1-PR8	1.97 (1.83–2.12)	0.08 (0.08–0.09)	0.11 (0.10–0.12)	0.17 (0.15–0.18)
H1N1-Ok-pdm	2.10 (1.93–2.27)	0.09 (0.08–0.10)	0.12 (0.11–0.13)	0.18 (0.17–0.20)

*FFU, focus-forming unit; H5N1-Ky, A/crow/Kyoto/53/04 (H5N1); H5N1-Eg, A/chicken/Egypt/CL6/07 (H5N1); H7N9, A/Anhui/1/23 (H7N9); H5N3, A/Duck/Hong Kong/820/80 (H5N3); H5N9, A/Turkey/Ontario/7732/66 (H5N9); H3N2, Clinical strain (H3N2); H1N1-PR8, A/Puerto Rico /8/1934 (H1N1); H1N1-Ok-pdm, A/Osaka/64/2009 (H1N1). †The elapsed time was used as the explanatory variable (x-axis), and the logarithmic virus titer was used as the explained variable (y-axis). A linear regression analysis with logarithmic link function was performed for each virus to create a curve of regression (Appendix Figure 2). Because the detection limit of each influenza virus titer was 10^1^ FFUs, the X value (when the Y value of the regression curve was 1.0) was used as the survival times. ‡The half-life of each virus was calculated from the slope of each regression curve when the amount of virus remaining on the surface was 2, 3, or 4 log_10_ FFUs.

### Disinfectant Effectiveness against Influenza Virus (In Vitro Evaluations)

All influenza viruses were completely inactivated (below the detection limit) within 15 seconds by treatment with 40%, 60%, or 80% EA or 70% IPA (log reductions in titers were >4). However, all viruses were not inactivated by 20% EA (log reduction <1). Of note, although all subtypes except for H5N1 were completely inactivated within 15 seconds by 36% EA (log reduction >4), the disinfectant effectiveness of 36% EA against H5N1-Ky and H5N1-Eg was substantially low (log reduction <3) (Table 3; Appendix Table 1).

**Table 3:** Results of in vitro evaluations of disinfectant effectiveness against various subtypes of influenza viruses*

Disinfectant	log reduction, mean							
H5N1-Ky	H5N1-Eg	H7N9	H5N3	H5N9	H3N2	H1N1-PR8	H1N1-Ok-pdm
80% EA	>4.00	>4.00	>4.00	>4.00	>4.00	>4.00	>4.00	>4.00
60% EA	>4.00	>4.00	>4.00	>4.00	>4.00	>4.00	>4.00	>4.00
40% EA	>4.00	>4.00	>4.00	>4.00	>4.00	>4.00	>4.00	>4.00
36% EA	2.57	1.77	>4.00	>4.00	>4.00	>4.00	>4.00	>4.00
34% EA	0.29	0.28	1.60	1.54	1.54	1.46	1.53	1.48
32% EA	0.11	0.16	0.23	0.20	0.27	0.23	0.23	0.21
20% EA	0.03	0.04	0.10	0.10	0.13	0.04	0.09	0.04
70% IPA	>4.00	>4.00	>4.00	>4.00	>4.00	>4.00	>4.00	>4.00
0.2% CHG	0.43	0.42	0.58	0.54	0.66	0.52	0.55	0.65
1.0% CHG	1.05	1.35	1.17	1.54	1.59	1.47	1.52	1.53
0.05% BAC	1.66	1.63	1.70	2.03	2.48	1.88	2.00	2.15
0.2% BAC	3.13	3.11	2.97	3.35	3.50	3.27	2.95	3.42

*Reaction time with disinfectant was 15 seconds. Detailed data are presented in Appendix Table 1. Log reduction value was calculated to evaluate disinfectant effectiveness under each condition and was expressed as mean. In addition, log reduction value of the condition wherein the virus was inactivated below the measurement limit (10^1.6^ FFUs) was 4 or more and was expressed as >4.00. BAC, benzalkonium chloride; CHG, chlorhexidine gluconate; EA, ethyl alcohol; H5N1-Ky, A/crow/Kyoto/53/04 (H5N1); H5N1-Eg, A/chicken/Egypt/CL6/07 (H5N1); H7N9, A/Anhui/1/23 (H7N9); H5N3, A/Duck/Hong Kong/820/80 (H5N3); H5N9, A/Turkey/Ontario/7732/66 (H5N9); H3N2, Clinical strain (H3N2); H1N1-PR8, A/Puerto Rico /8/1934 (H1N1); H1N1-Ok-pdm, A/Osaka/64/2009 (H1N1); IPA, isopropanol.

CHG and BAC were less effective than EA and IPA. The effectiveness of 0.2% GCH was low for all influenza viruses (log reduction <1), and 1.0% GCH was more effective than 0.2% GCH. BAC was more effective against all influenza viruses than CHG, and its effectiveness increased with increasing concentrations and disinfection times. In particular, treatment with 0.2% BAC for 15 seconds showed a log reduction value of >2.5, whereas the log reduction was >3.5 after a 60-second treatment (Table 3; Appendix Table 1).

### Effectiveness of Disinfectants against Influenza Virus on Human Skin (Ex Vivo Evaluations)

All viruses were completely inactivated on the skin surface within 15 seconds after treatment with 40%, 60%, or 80% EA or 70% IPA (log reduction >4). However, all viruses were barely inactivated by 20% EA (log reduction <1). Of note, although all subtypes except H5N1 were completely inactivated within 15 seconds by 36% EA (log reduction >4), the disinfectant effectiveness of 36% EA against H5N1-Ky and H5N1-Eg was substantially lower (log reduction <2) (Table 4; Appendix Table 2).

**Table 4:** Results of ex vivo evaluations of disinfectant effectiveness of disinfectants against various subtypes of influenza viruses on the surface of human skin*

Disinfectant	log reduction, mean							
H5N1-Ky	H5N1-Eg	H7N9	H5N3	H5N9	H3N2	H1N1-PR8	H1N1-Ok-pdm
80% EA	>4.00	>4.00	>4.00	>4.00	>4.00	>4.00	>4.00	>4.00
60% EA	>4.00	>4.00	>4.00	>4.00	>4.00	>4.00	>4.00	>4.00
40% EA	>4.00	>4.00	>4.00	>4.00	>4.00	>4.00	>4.00	>4.00
36% EA	1.71	1.61	>4.00	>4.00	>4.00	>4.00	>4.00	>4.00
34% EA	1.39	1.32	2.59	2.56	2.54	2.26	2.46	2.61
32% EA	1.17	1.14	2.20	2.18	2.18	2.31	2.21	2.18
20% EA	0.84	0.82	0.04	0.84	0.81	0.65	0.83	0.82
70% IPA	>4.00	>4.00	>4.00	>4.00	>4.00	>4.00	>4.00	>4.00
0.2% CHG	1.16	1.12	0.88	1.16	0.95	0.89	1.05	0.94
1.0% CHG	2.76	2.68	3.02	2.90	2.95	2.78	2.98	2.95
0.05% BAC	1.81	1.74	1.78	1.80	1.78	1.66	1.86	1.84
0.2% BAC	3.10	3.02	3.26	3.12	3.09	2.73	2.98	3.16

*Reaction time with disinfectant was 15 seconds. Detailed data are presented in Appendix Table 2. Log reduction value was calculated to evaluate disinfectant effectiveness under each condition and was expressed as mean. In addition, the log reduction value of the condition wherein the virus was inactivated below the measurement limit (10^1^ FFUs) was 4 or more and was expressed as >4.00. BAC, benzalkonium chloride; CHG, chlorhexidine gluconate; EA, ethyl alcohol; H5N1-Ky, A/crow/Kyoto/53/04 (H5N1); H5N1-Eg, A/chicken/Egypt/CL6/07 (H5N1); H7N9, A/Anhui/1/23 (H7N9); H5N3, A/Duck/Hong Kong/820/80 (H5N3); H5N9, A/Turkey/Ontario/7732/66 (H5N9); H3N2, Clinical strain (H3N2); H1N1-PR8, A/Puerto Rico /8/1934 (H1N1); H1N1-Ok-pdm, A/Osaka/64/2009 (H1N1); IPA, isopropanol.

CHG and BAC were less effective than EA and IPA. The effectiveness of CHG against all influenza viruses on human skin was higher than the in vitro disinfection effectiveness, and it increased as the CHG concentration and disinfection time increased. In particular, treatment with 1.0% CPG for 15 seconds showed log-reduction values of >2, and treatment with 1.0% CPG for 60 seconds showed log-reduction values of >2.5. In addition, BAC was more effective against all influenza viruses than CHG, and its effectiveness increased with increasing concentrations and disinfection times. Specifically, the log-reduction values after treatment with 0.2% BAC for 15 seconds and 60 seconds were >2.5 and >3.0 (Table 4; Appendix Table 2).

### Stability of Recombinant Viruses on Plastic and Human Skin Surfaces

Although all recombinant viruses (except rH5N3-H5N1-NA) became inactive on the plastic surface within 10 hours, rH5N3-H5N1-NA survived considerably longer than 10 hours. In addition, the titer of rH5N3-H5N1-NA remaining on the plastic surface was significantly higher than that of the other recombinant viruses at most time points (Figure 1, panel C). The survival times of the recombinant viruses (except for rH5N3-H5N1-NA) were ≈8 hours. For example, the survival time of rH5N1-H5N3NA was 8.15 (95% CI 6.86–9.62) hours. In contrast, the survival time of rH5N3-H5N1-NA was 23.68 (95% CI 21.68–26.25) hours, which was significantly longer than survival time of the other recombinant viruses tested (Table 5; Figure 2, panel E). Furthermore, half-lives showed the same tendency as survival times, and the half-life of rH5N3-H5N1-NA was more than twice that of other recombinant viruses (Table 5; Figure 2, panel F).

**Table 5:** Survival times and half-lives of various recombinant viruses on a plastic surface*

Subtype†	Median survival time (95% CI), h‡	Median half-life (95% CI), h§		
4 (log_10_ FFU)	3 (log_10_ FFU)	2 (log_10_ FFU)
rH5N1-H5N3-NA	8.15 (6.86–9.62)	0.41 (0.34–0.51)	0.55 (0.45–0.68)	0.82 (0.67–1.02)
rH5N1-H5N3-HA	8.17 (6.88–9.63)	0.41 (0.34–0.51)	0.55 (0.45–0.68)	0.83 (0.68–1.02)
rH5N3-H5N1-NA	23.68 (21.26–26.25)	1.14 (1.02–1.28)	1.52 (1.36–1.71)	2.28 (2.04–2.57)
rH5N3-H5N1-NS	7.74 (6.59–9.03)	0.39 (0.33–0.48)	0.53 (0.44–0.64)	0.79 (0.65–0.96)
rH5N3-H5N1-M	8.75 (7.52–10.11)	0.45 (0.38–0.54)	0.60 (0.50–0.72)	0.90 (0.75–1.08)
rH5N3-H5N1-HA	7.69 (6.59–8.93)	0.39 (0.33–0.47)	0.52 (0.44–0.63)	0.78 (0.65–0.95)

*FFU, focus-forming unit. †A/crow/Kyoto/53/04 (H5N1) recombined with the NA and HA genes of A/Duck/Hong Kong/820/80 (H5N3) are defined as rH5N1-H5N3-NA and rH5N1-H5N3-HA. In addition, A/Duck/Hong Kong/820/80 (H5N3) recombined into the NA, NS, M, and HA genes of A/crow/Kyoto/53/04 (H5N1) was defined as rH5N3-H5N1-NA, rH5N3-H5N1-NS, rH5N3-H5N1-M, rH5N3-H5N1-HA. ‡The elapsed time was used as the explanatory variable (x-axis), and the logarithmic virus titer was used as the explained variable (y-axis). A linear regression analysis with logarithmic link function was performed for each virus to create a curve of regression (Appendix Figure 3). Because the detection limit of each influenza virus titer was 10^1^ FFUs, the X value (when the Y value of the regression curve was 1.0) was used as the survival time. §The half-life of each virus was calculated from the slope of each regression curve when the amount of virus remaining on the surface was 2, 3, or 4 log_10_ FFUs.

Although all recombinant viruses (except rH5N3-H5N1-NA) became inactive on the human skin within 1.5 hours, rH5N3-H5N1-NA remained infective for considerably longer. Moreover, the titer of rH5N3-H5N1-NA remaining on the skin was significantly higher than that of other recombinant viruses at most time points (Figure 1, panel D). The survival times of recombinant viruses (except rH5N3-H5N1-NA) was ≈2.2 hours. For example, the survival time of rH5N1-H5N3NA was 2.04 (95% CI 1.79–2.31) hours. In contrast, the survival time of rH5N3-H5N1-NA was 4.65 (95% CI 3.94–5.43) hours, which was significantly longer than other recombinant viruses (Table 6; Figure 2, panel G). In addition, half-lives showed the same tendency as survival times, and the half-life of rH5N3-H5N1-NA was more than twice that of other recombinant viruses (Table 6; Figure 2, panel H).

**Table 6:** Survival time and half-lives of various recombinant viruses on the surface of human skin*

Subtype†	Median survival time (95% CI), h‡	Median half-life (95% CI), h‡		
4 (log_10_ FFU)	3 (log_10_ FFU)	2 (log_10_ FFU)
rH5N1-H5N3-NA	2.04 (1.79–2.31)	0.09 (0.08–0.10)	0.12 (0.10–0.14)	0.18 (0.15–0.21)
rH5N1-H5N3-HA	2.06 (1.77–2.37)	0.09 (0.08–0.11)	0.12 (0.10–0.14)	0.18 (0.15–0.21)
rH5N3-H5N1-NA	4.65 (3.94–5.43)	0.20 (0.17–0.25)	0.27 (0.23–0.33)	0.41 (0.35–0.49)
rH5N3-H5N1-NS	2.18 (1.83–2.55)	0.10 (0.08–0.12)	0.13 (0.11–0.16)	0.19 (0.16–0.23)
rH5N3-H5N1-M	2.22 (1.87–2.61)	0.10 (0.08–0.12)	0.13 (0.11–0.16)	0.19 (0.16–0.24)
rH5N3-H5N1-HA	2.16 (1.83–2.52)	0.09 (0.07–0.11)	0.13 (0.11–0.15)	0.19 (0.16–0.23)

*FFU, focus-forming unit. †A/crow/Kyoto/53/04 (H5N1) recombined with the NA and HA genes of A/Duck/Hong Kong/820/80 (H5N3) are defined as rH5N1-H5N3-NA and rH5N1-H5N3-HA, respectively. In addition, A/Duck/Hong Kong/820/80 (H5N3) recombined into the NA, NS, M, and HA genes of A/crow/Kyoto/53/04 (H5N1) was defined as rH5N3-H5N1-NA, rH5N3-H5N1-NS, rH5N3-H5N1-M, rH5N3-H5N1-HA. ‡The elapsed time was used as the explanatory variable (x-axis), and the logarithmic virus titer was used as the explained variable (y-axis). A linear regression analysis with logarithmic link function was performed for each virus to create a curve of regression (Appendix Figure 4). Since the detection limit of each influenza virus titer was 10^1^ FFUs, the X value (when the Y value of the regression curve was 1.0) was used as the survival time. §The half-life of each virus was calculated from the slope of each regression curve when the amount of virus remaining on the surface was 2, 3, or 4 log_10_ FFUs.

### Disinfectant Effectiveness of a Relatively Low EA Concentration against Recombinant Viruses

**Figure 3:**
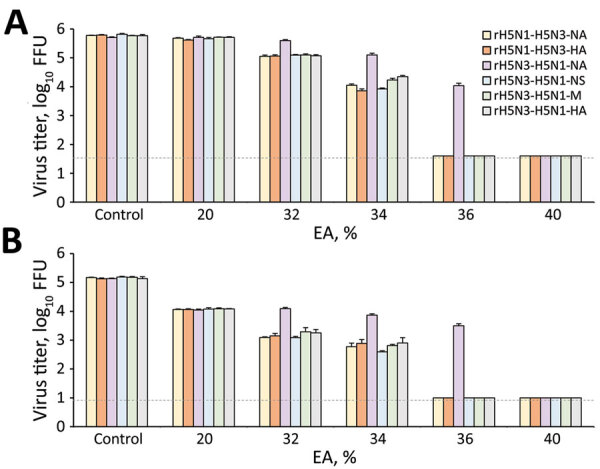
Effectiveness of disinfectants against various recombinant influenza viruses. A, B) In vitro (A) and ex vivo (B) evaluations were performed, and the residual viral titer after EA exposure is shown. The results are expressed as mean + SD. Dotted horizontal lines represent the detection limit titers. A/crow/Kyoto/53/04 (H5N1) was recombined with the neuraminidase or hemagglutinin gene of A/Duck/Hong Kong/820/80 (H5N3), and the recombinant viruses were designated as rH5N1-H5N3-NA and rH5N1-H5N3-HA. In addition, A/Duck/Hong Kong/820/80 (H5N3) was recombined with the neuraminidase, nonstructural protein, matrix protein, or hemagglutinin gene of A/crow/Kyoto/53/04 (H5N1), and the recombinant viruses were designated as rH5N3-H5N1-NA, rH5N3-H5N1-NS, rH5N3-H5N1-M, or rH5N3-H5N1-HA. log reductions were calculated to evaluate the effectiveness of disinfectants under different conditions (Appendix Table 3). EA, ethyl alcohol.

Both in vitro and ex vivo evaluations demonstrated that all recombinant viruses were completely inactivated within 15 seconds by treatment with >40% EA (log reduction >4). Furthermore, although all recombinant viruses (except rH5N3-H5N1-NA) were completely inactivated within 15 seconds by treatment with 36% EA (log reduction >4), 36% EA was substantially less effective against rH5N3-H5N1-NA (log reduction <2). Thus, rH5N3-H5N1-NA was resistant to relatively low EA concentrations (Figure 3; Appendix Table 3).

## Discussion

Previous studies have suggested that the stability of AIVs might vary among subtypes, but the details remain unknown (*20*–*22*,*25*). In this study, we first evaluated the stability (survival time and half-life) of several influenza subtypes on plastic and human skin surfaces and clarified the differences in their stability. No significant differences were observed in the survival times and half-lives of most subtypes. However, the survival times and half-lives of 2 different H5N1 strains (H5N1-Ky and H5N1-Eg) on plastic and skin surfaces were approximately twice as long as those of the other subtypes tested, indicating that the H5N1 subtype had significantly higher stability. These findings suggest that the H5N1 subtype poses a higher risk for contact transmission than other subtypes. Specifically, the higher stability of the H5N1 subtype might be a reason that among AIVs, the H5N1 subtype is often transmitted from birds to humans. In addition, because the 4-hour survival time of the H5N1 subtype on human skin increases the risk for viral invasion into the body or for transmission from the skin to other surfaces, appropriate hand hygiene practices are especially vital (compared with other subtypes) for preventing contact transmission of this subtype. Furthermore, the survival times revealed in this study will help determine the interval during which contact transmission could occur and how contact transmission might be established.

Next, we evaluated the effectiveness of disinfectants against influenza viruses on the skin surface by using our ex vivo evaluation model that reproduced actual hand hygiene condition and elucidated the differences in disinfectant efficacy against different subtypes (*26*–*28*). All viruses on the skin surface were completely inactivated by exposure to alcohol-based disinfectants (high concentrations of EA or IPA) for 15 seconds. In addition, most viruses on the skin surface were completely inactivated by exposure to 36% EA for 15 seconds, but the H5N1 subtype was not. These findings reveal that the H5N1 subtype was more resistant to EA than other subtypes and that the effectiveness of relatively low EA concentrations (36% wt/wt or 43% vol/vol) against the H5N1 subtype was lower. Therefore, to control contact transmission of the H5N1 subtype, disinfectants with appropriate EA concentrations, as proposed by the World Health Organization (>52% wt/wt or >60% vol/vol), should be used (*41*). Although low-level disinfectants such as BAC and CHG were much less effective than alcohol-based disinfectants, high concentrations of low-level disinfectants (i.e., 0.2% BAC or 1.0% CHG) were relatively effective against all influenza viruses on the skin surface. These results suggest that high concentrations of BAC-based and CHG-based disinfectants might be applicable for hand hygiene targeting influenza viruses as an alternative to alcohol-based disinfectants, although additional studies are needed to validate this possibility.

Finally, we tried to elucidate the genetic mechanisms responsible for differences in stability and disinfectant effectiveness among subtypes by using different recombinant viruses. The stability of all recombinant viruses tested (except rH5N3-H5N1-NA) on plastic and human skin surfaces was similar to that of all influenza viruses studied (except H5N1). Moreover, the survival time and half-life of rH5N3-H5N1-NA (a recombinant H5N3 virus with the NA gene of an H5N1 virus) on the plastic and human skin surfaces were approximately twice as long as other recombinant viruses, and it had the same stability as the H5N1 subtype (H5N1-Ky and H5N1-Eg). While evaluating the effectiveness of disinfectants, we found that although all recombinant viruses tested (except rH5N3-H5N1-NA) were completely inactivated by exposure to 36% EA for 15 seconds, only rH5N3-H5N1-NA was not significantly inactivated by exposure to 36% EA, and it had the same EA resistance as the H5N1 subtype. Those results strongly suggest that the higher stability and EA resistance of the H5N1 subtype might depend on NA, a spike protein. Although several studies have focused on the relationship between the NA segment and virulence (*42*,*43*), to the best of our knowledge, no study has focused on the relationship between the NA segment and stability. Future studies focusing on the NA segment are expected to elucidate factors that determine the stability and help identify subtypes with high stability and a high risk for contact transmission.

The first limitation of our study is that we used an ex vivo evaluation model in this study using human skin samples collected during forensic autopsies, because the application of highly pathogenic viruses (such as the H5N1 subtype) on the skin of humans is dangerous. At this stage, we tentatively conclude that virus survival time would not substantially differ between autopsy skin specimens and live human skin or between the different autopsy specimens. However, improving measurement accuracy, increasing the number of cumulative measurement samples, and more thorough evaluation of skin properties might elucidate the properties of skin samples and donor factors that affect virus survival. Second, we analyzed virus stability by mixing virus and PBS in this study. The use of solvents other than PBS (e.g., cell culture medium or human upper respiratory tract–derived mucus) might affect the residual virus titer on the surface and the analysis results. Furthermore, the evaluation was performed in a controlled environment (25°C and 45%–55% relative humidity); however, changes in temperature and humidity might have an effect on virus stability. Finally, this study revealed that the NA proteins in the influenza virus might contribute to the high stability of the H5N1 subtype, but the properties of the NA proteins that affect virus stability were not elucidated. In the future, preparing recombinant viruses with various NA proteins and clarifying the properties of NA that affect virus stability will be necessary.

In conclusion, we found that the H5N1 subtype had a higher risk for contact transmission because of its higher stability on plastic and skin surfaces and higher resistance to EA than other subtypes. Therefore, the optimal infection control methods may differ for each subtype. Our findings also suggest that these characteristics might depend on the NA protein. 

Appendix Additional information about higher viral stability and ethanol resistance of avian influenza A (H5N1) virus on human skin.
