# Supplementary material for: Higher Viral Stability and Ethanol Resistance of Avian Influenza A(H5N1) Virus on Human Skin
Source: Emerg Infect Dis. 2022 Mar;28(3):639–49. doi: 10.3201/eid2803.211752 (PMC8888214; doi:10.3201/eid2803.211752)
Supplement: Appendix — Additional information about higher viral stability and ethanol resistance of avian influenza A (H5N1) virus on human skin. [file 21-1752-Techapp-s1.pdf]

Article DOI: https://doi.org/10.3201/eid2803.211752

Higher Viral Stability and Ethanol Resistance of Avian Influenza A (H5N1) Virus on Human Skin

Appendix

Detailed Methods

Cell Culture

Madin–Darby canine kidney (MDCK) cells were purchased from RIKEN BioResource Center Cellbank (Ibaraki, Japan). They were cultured in minimum essential medium (Sigma-Aldrich, https://www.sigmaaldrich.com) supplemented with 10% fetal bovine serum and standard antibiotics (penicillin and streptomycin).

Viruses

A/crow/Kyoto/53/04(H5N1) (H5N1-Ky), A/chicken/Egypt/CL6/07(H5N1) (H5N1-Eg), A/Anhui/1/23(H7N9) (H7N9), A/Duck/Hong Kong/820/80(H5N3) (H5N3), A/Turkey/Ontario/7732/66(H5N9) (H5N9), a clinical H3N2 strain (H3N2), A/Puerto Rico/8/1934(H1N1) (H1N1-PR8), and A/Osaka/64/2009 (H1N1-Ok-pdm) were propagated in 9-day-old embryonic chicken eggs. The allantoic fluids were precleared by centrifugation at 3300 × *g* for 30 min and subsequent filtration through a 0.45-µm filter to remove impurities in the urinary fluid in advance. Finally, virus in the allantoic fluid was purified through a 20% (w/v) sucrose cushion in phosphate-buffered saline (PBS) by ultracentrifugation at 28,000 rpm for 2.5

h at 4°C in a Beckman SW28 rotor. The virus pellets were suspended in PBS and stored at −80°C as a working stock (*1*). The viral titers were measured by performing focus-forming assay with MDCK cells and expressed as the number of focus-forming units (FFUs). At 12 h post-infection, the MDCK cells were fixed with PBS containing 4% paraformaldehyde and 0.1% Triton X-100 for 30 min at room temperature (25°C). Viral antigens were detected by staining the cells with a rabbit polyclonal antibody, diluted 1:1000 in PBS (−) containing 1% bovine serum albumin. The antibody is produced against A/duck/Hong Kong/342/78 (H5N2) and recognizes the viral nucleoprotein (NP) and matrix protein 1 (M1). Viral proteins were detected using an Alexa Fluor 488-conjugated secondary antibody (diluted 1:500 in PBS) (ThermoFisher Scientific, https://www.thermofisher.com), and cells labeled for the viral antigen were counted under a fluorescence microscope (*2*).

A/Anhui/1/2013 (H7N9) was kindly provided by Dr. Shu Yuelong (World Health Organization [WHO] Collaborating Center for Reference and Research on Influenza, Chinese Center for Disease Control and Prevention, Beijing, China) through the National Institute of Infectious Diseases, Japan. We executed a Standard Material Transfer Agreement 2 (SMTA2) with the WHO to receive the H7N9 virus, which is a pandemic influenza preparedness biologic material. Experiments performed with the avian influenza virus were conducted at Kyoto Prefectural University of Medicine under Biosafety Level 3+ conditions (approved by the Ministry of Agriculture, Forestry and Fisheries of Japan).

Generation of Recombinant H5N3 and H5N1 Viruses

A recombinant H5N3 virus was generated by using a reverse-genetics system by recombining each H5N1-Ky (NA, NS, M, HA) gene with H5N3 (*3*–*6*). We prepared 8 types of plasmids for amplifying viral gene segments. One of the 8 plasmids used to synthesize a viral

gene segment served as the H5N1-Ky viral gene segment plasmid, whereas the remaining plasmids were derived from H5N3, which generated a recombinant H5N3 virus containing specific H5N1-Ky gene segments. In addition, a pCAGGS expression plasmid that expresses proteins (PB2, PB1, WSN, PA, and NP) required for the transcription and replication of viral gene segments was introduced into 293T cells (human fetal kidney epithelial cells). Seven genome segments of H5N3 and the NA, NS, M, or HA genes of H5N1-Ky were constructed by reverse transcription PCR-based amplification, as reported previously (*3–6*). Each plasmid was introduced into cells, and acetylated trypsin (5 µg/mL) (Merck, https://www.merck.com) was added to the plates 1 or 4 days post-transfection. At 7 days post-transfection, the culture supernatants were collected and injected into 9-day-old chicken eggs. The allantoic fluid was collected 3 days post-injection and purified as described previously, followed by titration on MDCK cells to measure the virus titers. All recombinant H5N3 viruses were confirmed by sequencing the viral genes.

The same methods were applied to produce recombinant H5N1-Ky. Briefly, H5N1-Ky was recombined with the NA and HA genes of an H5N3 virus, and the recombinant viruses were designated as rH5N1-H5N3-NA and rH5N1-H5N3-HA. In addition, H5N3 was recombined with the NA, NS, M, and HA genes of H5N1-Ky and designated as rH5N3-H5N1-NA, rH5N3-H5N1-NS, rH5N3-H5N1-M, and rH5N3-H5N1-HA.

Construction of a Skin Model to Evaluate Virus Stability and Disinfectant Effectiveness

Human skin was collected from forensic autopsy specimens obtained from the Department of Forensic Medicine, Kyoto Prefectural University of Medicine. Abdominal skin autopsy specimens from persons 20–70 years of age, obtained ≈1 day after death, were cut into

squares with approximate dimensions of 4 cm × 8 cm. Autopsy specimens in which the skin was considerably damaged by burning or drowning were excluded (*7*,*8*).

Using the autopsy skin specimens, an ex vivo model was developed to evaluate the stability of different viruses on the surface of human skin and the effectiveness of different disinfectants against viruses on human skin. This model enabled long-term culturing of skin specimens to avoid deterioration of the skin samples because of drying. Skin from which the panniculus adiposus had been removed was washed with PBS and placed in a culture insert (Corning Inc., https://www.corning.com) on a membrane of pore size 8.0 μm. The culture inserts were placed in six-well plates containing 1.0 mL of Dulbecco's modified Eagle's medium (DMEM; Sigma-Aldrich) (*7*,*9*).

Human skin (particularly the epidermis) is characterized by slower deterioration after death than other organs, and the collected skin can be used for grafting even 24 hours after death (*10*,*11*). Moreover, previous studies, in which an ex vivo model was constructed using skin within 36 hours of excision, reported that the post-extraction skin retained its physiologic function relatively well with no change in cell viability after 14 days in culture (*12*,*13*). Therefore, this evaluation model, using skin collected from autopsy specimens ≈1 day after death, could preserve skin functions and successfully model the in vivo conditions.

Evaluating the Virus Stability on Plastic and Human Skin Surfaces

Virus survival was evaluated on plastic and human skin surfaces. Virus solutions (2.0×10^5 FFUs in 2 μL PBS) were applied on the surface of plastic or human skin (the constructed evaluation model). Each sample was incubated in a controlled environment (25°C, 45%–55% relative humidity) for 0–24 hours. The virus remaining on the surface was then collected in 1.0 mL of DMEM and titrated (*7*,*14*–*17*). The detection limit for the titer of the virus remaining on

the surface was 10^1 FFUs. Survival time is defined as the time until the virus on the surface is no longer detected. We performed 3 independent experiments for each condition, and the results are expressed as mean $\pm$ standard deviation of the mean.

Previous studies have reported that a maximum viral titer concentration in the upper respiratory tract–derived body fluids of patients with influenza infection is $\approx 2.0 \times 10^6$ FFUs/mL or $TCID_{50}$/mL (*18*,*19*). When 10–100 µL of the infectious body fluid is deposited on a surface, the maximum absolute viral titer on the surface is $\approx 2.0 \times 10^5$ FFUs. In this study, we aimed to assess the risk for contact transmission, and the risk for contact transmission should not be underestimated. Therefore, we evaluated the stability and disinfection efficacy under the condition that the virus adhered to the skin surface with 2.0×10^5 FFUs, which is the upper limit of the absolute titer.

Evaluating the Effectiveness of Disinfectants against Viruses In Vitro

The effectiveness of several disinfectants was evaluated at different concentrations. The effectiveness of EA (Nacalai Tesque, https://www.nacalai.co.jp) was tested at concentrations of 20%, 32%, 34%, 36%, 40%, 60%, and 80% (w/w). IPA (Nacalai Tesque) was tested at a concentration of 70% (w/w). CHG was tested at concentrations of 0.2% and 1.0% (w/v) (Saraya, https://saraya.world). BAC (Yakuhan Pharmaceutical, https://www.nichiiko.co.jp/yakuhan) was tested at concentrations of 0.05% and 0.2% (w/v).

In a 1.5-mL tube, 5 µL of PBS containing either avian or human influenza virus (4.0×10^5 FFUs in 5 µL PBS) was mixed with 95 µL of various disinfectants for 15 or 60 seconds. Subsequently, the resulting solutions were neutralized with 900 µL of Soybean–Casein Digest Broth prepared with Lecithin & Polysorbate 80 'DAIGO' (SCLDP) medium. Thereafter, 3 mL of DMEM was added to the neutralized solution, and the remaining viral titers were measured

(*9*,*20*–*22*). The detection limit for the virus titers was $10^{1.6}$ FFUs. To evaluate the disinfectant effectiveness under each condition, logarithmic reductions of the viral titers were calculated, with normalization to the PBS control titers. Three independent experiments were performed for each condition, and the results are expressed as mean ± standard deviation of the mean.

Evaluating the Effectiveness of Disinfectants against Viruses on the Human Skin Surface (Ex Vivo Evaluations)

The same disinfectants used for in vitro evaluations were used for ex vivo evaluations. Each virus solution (2.0×10^5 FFUs in 2 µL PBS) was applied to the surface of human skin (the constructed evaluation model). Each skin sample was then incubated for 15 minutes at 25°C under 45%–55% relative humidity, to dry the viral mixture on the skin completely. Subsequently, each skin sample surface was immersed in 1 mL of the disinfectant for 15 or 60 seconds, and then air-dried for 5 minutes. After drying, the remaining viruses on the skin were recovered with 250 µL of SCDLP and 750 µL of DMEM, and the remaining viral load was measured. The detection limit for the virus titers was 10^1 FFUs (*7*,*9*). To determine the effectiveness of the disinfectants under each condition, logarithmic reductions of the virus titers were calculated, with normalization to the PBS control titers. Three independent experiments were performed for each condition, and the results are expressed as mean ± standard deviation of the mean.

Statistical Analysis

GraphPad Prism 9 software (GraphPad, Inc, La Jolla, CA) was used to analyze the data. The elapsed time was used as the explanatory variable (x-axis), and the logarithmic virus titer was used as the explained variable (y-axis). Least-squares linear-regression analysis was performed using a logarithmic link function to create regression curves for both viruses. As the

detection limit of each IFV titer was 10^1 FFUs, the X value (when the Y value of the regression curve was 1.0) was used as the survival time. The half-life of each virus was calculated from the slope of each regression curve when the amount of virus remaining on the surface was 2, 3, or 4 Log_{10} FFUs (*7*,*15*).

References

1. Daidoji T, Watanabe Y, Ibrahim MS, Yasugi M, Maruyama H, Masuda T, et al. Avian influenza virus infection of immortalized human respiratory epithelial cells depends upon a delicate balance between hemagglutinin acid stability and endosomal pH. J Biol Chem. 2015;290:10627–42. PubMed https://doi.org/10.1074/jbc.M114.611327

2. Hirose R, Nakaya T, Naito Y, Daidoji T, Watanabe Y, Yasuda H, et al. Mechanism of human influenza virus RNA persistence and virion survival in feces: mucus protects virions from acid and digestive juices. J Infect Dis. 2017;216:105–9. PubMed https://doi.org/10.1093/infdis/jix224

3. Basler CF, Reid AH, Dybing JK, Janczewski TA, Fanning TG, Zheng H, et al. Sequence of the 1918 pandemic influenza virus nonstructural gene (NS) segment and characterization of recombinant viruses bearing the 1918 NS genes. Proc Natl Acad Sci U S A. 2001;98:2746–51. PubMed https://doi.org/10.1073/pnas.031575198

4. Fodor E, Devenish L, Engelhardt OG, Palese P, Brownlee GG, García-Sastre A. Rescue of influenza A virus from recombinant DNA. J Virol. 1999;73:9679–82. PubMed https://doi.org/10.1128/JVI.73.11.9679-9682.1999

5. Tumpey TM, García-Sastre A, Mikulasova A, Taubenberger JK, Swayne DE, Palese P, et al. Existing antivirals are effective against influenza viruses with genes from the 1918 pandemic virus. Proc Natl Acad Sci U S A. 2002;99:13849–54. PubMed https://doi.org/10.1073/pnas.212519699

6. Neumann G, Watanabe T, Ito H, Watanabe S, Goto H, Gao P, et al. Generation of influenza A viruses entirely from cloned cDNAs. Proc Natl Acad Sci U S A. 1999;96:9345–50. PubMed https://doi.org/10.1073/pnas.96.16.9345

7. Hirose R, Ikegaya H, Naito Y, Watanabe N, Yoshida T, Bandou R, et al. Survival of SARS-CoV-2 and influenza virus on the human skin: Importance of hand hygiene in COVID-19. Clin Infect Dis. 2020. PubMed

8. Akutsu T, Ikegaya H, Watanabe K, Miyasaka S. Immunohistochemical staining of skin-expressed proteins to identify exfoliated epidermal cells for forensic purposes. Forensic Sci Int. 2019;303:109940. PubMed https://doi.org/10.1016/j.forsciint.2019.109940

9. Hirose R, Bandou R, Ikegaya H, Watanabe N, Yoshida T, Daidoji T, et al. Disinfectant effectiveness against SARS-CoV-2 and influenza viruses present on human skin: model-based evaluation. Clin Microbiol Infect. 2021;27:1042.e1–1042.e4. **PMID 33901670**

10. Hirel B, Watier E, Chesne C, Patoux-Pibouin M, Guillouzo A. Culture and drug biotransformation capacity of adult human keratinocytes from post-mortem skin. Br J Dermatol. 1996;134:831–6. PubMed https://doi.org/10.1111/j.1365-2133.1996.tb06311.x

11. Boekema BK, Boekestijn B, Breederveld RS. Evaluation of saline, RPMI and DMEM/F12 for storage of split-thickness skin grafts. Burns. 2015;41:848–52. PubMed https://doi.org/10.1016/j.burns.2014.10.016

12. Corzo-León DE, Munro CA, MacCallum DM. An *ex vivo* human skin model to study superficial fungal infections. Front Microbiol. 2019;10:1172. PubMed https://doi.org/10.3389/fmicb.2019.01172

13. Corzo-León DE, MacCallum DM, Munro CA. Host responses in an *Ex Vivo* human skin model challenged with *Malassezia sympodialis.* Front Cell Infect Microbiol. 2021;10:561382. PubMed https://doi.org/10.3389/fcimb.2020.561382

14. Oxford J, Berezin EN, Courvalin P, Dwyer DE, Exner M, Jana LA, et al. The survival of influenza A(H1N1)pdm09 virus on 4 household surfaces. Am J Infect Control. 2014;42:423–5. PubMed https://doi.org/10.1016/j.ajic.2013.10.016

15. Hirose R, Ikegaya H, Naito Y, Watanabe N, Yoshida T, Bandou R, et al. Reply to Gracely. Clin Infect Dis. 2021;73:e854–6. PubMed https://doi.org/10.1093/cid/ciab023

16. Chin AWH, Chu JTS, Perera MRA, Hui KPY, Yen HL, Chan MCW, et al. Stability of SARS-CoV-2 in different environmental conditions. Lancet Microbe. 2020;1:e10. PubMed https://doi.org/10.1016/S2666-5247(20)30003-3

17. van Doremalen N, Bushmaker T, Morris DH, Holbrook MG, Gamble A, Williamson BN, et al. Aerosol and surface stability of SARS-CoV-2 as compared with SARS-CoV-1. N Engl J Med. 2020;382:1564–7. PubMed https://doi.org/10.1056/NEJMc2004973

18. Branche AR, Walsh EE, Formica MA, Falsey AR. Detection of respiratory viruses in sputum from adults by use of automated multiplex PCR. J Clin Microbiol. 2014;52:3590–6. PubMed https://doi.org/10.1128/JCM.01523-14

19. Ng EK, Cheng PK, Ng AY, Hoang TL, Lim WW. Influenza A H5N1 detection. Emerg Infect Dis. 2005;11:1303–5. PubMed https://doi.org/10.3201/eid1108.041317

20. Gehrke C, Steinmann J, Goroncy-Bermes P. Inactivation of feline calicivirus, a surrogate of norovirus (formerly Norwalk-like viruses), by different types of alcohol in vitro and in vivo. J Hosp Infect. 2004;56:49–55. PubMed https://doi.org/10.1016/j.jhin.2003.08.019

21. Eggers M, Eickmann M, Kowalski K, Zorn J, Reimer K. Povidone-iodine hand wash and hand rub products demonstrated excellent in vitro virucidal efficacy against Ebola virus and modified vaccinia virus Ankara, the new European test virus for enveloped viruses. BMC Infect Dis. 2015;15:375. PubMed https://doi.org/10.1186/s12879-015-1111-9

22. Eggers M, Eickmann M, Zorn J. Rapid and effective virucidal activity of povidone-iodine products against Middle East respiratory syndrome coronavirus (MERS-CoV) and Modified Vaccinia Virus Ankara (MVA). Infect Dis Ther. 2015;4:491–501. PubMed https://doi.org/10.1007/s40121-015-0091-9

Appendix Table 1. Results of in vitro evaluations of disinfectant effectiveness against various subtypes of influenza viruses*

	Log reduction, mean ± standard deviation															
	H5N1-Ky		H5N1-Eg		H7N9		H5N3		H5N9		H3N2		H1N1-PR8		H1N1-Ok-pdm	
Disinfectant	15s	60s	15s	60s	15s	60s	15s	60s	15s	60s	15s	60s	15s	60s	15s	60s
80% EA	>4.00	>4.00	>4.00	>4.00	>4.00	>4.00	>4.00	>4.00	>4.00	>4.00	>4.00	>4.00	>4.00	>4.00	>4.00	>4.00
60% EA	>4.00	>4.00	>4.00	>4.00	>4.00	>4.00	>4.00	>4.00	>4.00	>4.00	>4.00	>4.00	>4.00	>4.00	>4.00	>4.00
40% EA	>4.00	>4.00	>4.00	>4.00	>4.00	>4.00	>4.00	>4.00	>4.00	>4.00	>4.00	>4.00	>4.00	>4.00	>4.00	>4.00
36% EA	2.57 ± 0.88	>4.00	1.77 ± 0.08	>4.00	>4.00	>4.00	>4.00	>4.00	>4.00	>4.00	>4.00	>4.00	>4.00	>4.00	>4.00	>4.00
34% EA	0.29 ± 0.07	3.23 ± 0.11	0.28 ± 0.09	3.08 ± 0.14	1.60 ± 0.21	>4.00	1.54 ± 0.10	>4.00	1.54 ± 0.03	>4.00	1.46 ± 0.08	>4.00	1.53 ± 0.27	>4.00	1.48 ± 0.06	>4.00
32% EA	0.11 ± 0.06	1.84 ± 0.14	0.16 ± 0.05	1.81 ± 0.11	0.23 ± 0.06	>4.00	0.20 ± 0.03	>4.00	0.27 ± 0.04	>4.00	0.23 ± 0.04	>4.00	0.23 ± 0.06	>4.00	0.21 ± 0.03	>4.00
20% EA	0.03 ± 0.07	0.03 ± 0.06	0.04 ± 0.03	0.03 ± 0.02	0.10 ± 0.05	0.04 ± 0.04	0.10 ± 0.08	0.03 ± 0.03	0.13 ± 0.07	0.07 ± 0.05	0.04 ± 0.04	0.04 ± 0.06	0.09 ± 0.02	0.04 ± 0.03	0.04 ± 0.04	0.05 ± 0.02
70% IPA	>4.00	>4.00	>4.00	>4.00	>4.00	>4.00	>4.00	>4.00	>4.00	>4.00	>4.00	>4.00	>4.00	>4.00	>4.00	>4.00
0.2% CHG	0.43 ± 0.20	0.38 ± 0.17	0.42 ± 0.05	0.37 ± 0.09	0.58 ± 0.12	0.54 ± 0.09	0.54 ± 0.11	0.53 ± 0.06	0.66 ± 0.03	0.65 ± 0.04	0.52 ± 0.19	0.55 ± 0.14	0.55 ± 0.12	0.56 ± 0.10	0.65 ± 0.03	0.67 ± 0.03
1.0% CHG	1.05 ± 0.34	1.30 ± 0.13	1.35 ± 0.10	1.29 ± 0.12	1.17 ± 0.45	1.91 ± 0.49	1.54 ± 0.05	2.15 ± 0.68	1.59 ± 0.09	2.66 ± 0.11	1.47 ± 0.12	2.05 ± 0.59	1.52 ± 0.12	1.90 ± 0.52	1.53 ± 0.03	2.39 ± 0.01
0.05% BAC	1.66 ± 0.21	3.00 ± 0.51	1.63 ± 0.18	3.02 ± 0.42	1.70 ± 0.07	2.77 ± 0.41	2.03 ± 0.38	3.03 ± 0.51	2.48 ± 0.15	3.29 ± 0.21	1.88 ± 0.17	3.06 ± 0.28	2.00 ± 0.33	2.93 ± 0.49	2.15 ± 0.10	3.36 ± 0.14
0.2% BAC	3.13 ± 0.08	3.75 ± 0.46	3.11 ± 0.06	3.73 ± 0.44	2.97 ± 0.46	3.80 ± 0.44	3.35 ± 0.25	>4.00	3.50 ± 0.10	>4.00	3.27 ± 0.14	>4.00	2.95 ± 0.25	>4.00	3.42 ± 0.05	>4.00

*Log reduction value was calculated to evaluate disinfectant effectiveness under each condition and was expressed as mean ± standard deviation. Additionally, the log reduction value of the condition wherein the virus was inactivated below the measurement limit ($10^{1.6}$ FFUs) was 4 or more and was expressed as >4.00. BAC, benzalkonium chloride; CHG, chlorhexidine gluconate; EA, ethyl alcohol; H5N1-Ky, A/crow/Kyoto/53/04 (H5N1); H5N1-Eg, A/chicken/Egypt/CL6/07 (H5N1); H7N9, A/Anhui/1/23 (H7N9); H5N3, A/Duck/Hong Kong/820/80 (H5N3); H5N9, A/Turkey/Ontario/7732/66 (H5N9); H3N2, Clinical strain (H3N2); H1N1-PR8, A/Puerto Rico /8/1934 (H1N1); H1N1-Ok-pdm, A/Osaka/64/2009 (H1N1); IPA, isopropanol.

Appendix Table 2. Results of ex vivo evaluations of disinfectant effectiveness of disinfectants against various subtypes of influenza viruses on the surface of human skin*

	Log reduction, mean ± standard deviation															
	H5N1-Ky		H5N1-Eg		H7N9		H5N3		H5N9		H3N2		H1N1-PR8		H1N1-Ok-pdm	
Disinfectant	15s	60s	15s	60s	15s	60s	15s	60s	15s	60s	15s	60s	15s	60s	15s	60s
80% EA	>4.00	>4.00	>4.00	>4.00	>4.00	>4.00	>4.00	>4.00	>4.00	>4.00	>4.00	>4.00	>4.00	>4.00	>4.00	>4.00
60% EA	>4.00	>4.00	>4.00	>4.00	>4.00	>4.00	>4.00	>4.00	>4.00	>4.00	>4.00	>4.00	>4.00	>4.00	>4.00	>4.00
40% EA	>4.00	>4.00	>4.00	>4.00	>4.00	>4.00	>4.00	>4.00	>4.00	>4.00	>4.00	>4.00	>4.00	>4.00	>4.00	>4.00
36% EA	1.71 ± 0.15	>4.00	1.61 ± 0.07	>4.00	>4.00	>4.00	>4.00	>4.00	>4.00	>4.00	>4.00	>4.00	>4.00	>4.00	>4.00	>4.00
34% EA	1.39 ± 0.06	2.92 ± 0.11	1.32 ± 0.01	2.79 ± 0.03	2.59 ± 0.09	>4.00	2.56 ± 0.12	>4.00	2.54 ± 0.08	>4.00	2.26 ± 0.26	>4.00	2.46 ± 0.06	>4.00	2.61 ± 0.02	>4.00
32% EA	1.17 ± 0.06	1.30 ± 0.02	1.14 ± 0.03	1.28 ± 0.06	2.20 ± 0.05	2.68 ± 0.08	2.18 ± 0.01	2.89 ± 0.21	2.18 ± 0.07	2.91 ± 0.20	2.31 ± 0.21	2.87 ± 0.13	2.21 ± 0.12	2.76 ± 0.29	2.18 ± 0.12	2.71 ± 0.22
20% EA	0.84 ± 0.04	1.00 ± 0.01	0.82 ± 0.06	0.99 ± 0.05	0.04 ± 0.06	1.01 ± 0.09	0.84 ± 0.04	1.04 ± 0.06	0.81 ± 0.02	1.08 ± 0.04	0.65 ± 0.05	0.81 ± 0.08	0.83 ± 0.05	1.08 ± 0.02	0.82 ± 0.06	1.04 ± 0.05
70% IPA	>4.00	>4.00	>4.00	>4.00	>4.00	>4.00	>4.00	>4.00	>4.00	>4.00	>4.00	>4.00	>4.00	>4.00	>4.00	>4.00
0.2% CHG	1.16 ± 0.03	1.32 ± 0.05	1.12 ± 0.05	1.28 ± 0.04	0.88 ± 0.22	1.00 ± 0.09	1.16 ± 0.05	1.28 ± 0.04	0.95 ± 0.20	1.19 ± 0.12	0.89 ± 0.16	0.82 ± 0.14	1.05 ± 0.05	1.25 ± 0.01	0.94 ± 0.07	1.12 ± 0.13
1.0% CHG	2.76 ± 0.04	3.09 ± 0.07	2.68 ± 0.06	3.05 ± 0.03	3.02 ± 0.15	3.06 ± 0.02	2.90 ± 0.11	3.00 ± 0.13	2.95 ± 0.26	3.01 ± 0.14	2.78 ± 0.23	2.85 ± 0.01	2.98 ± 0.38	3.02 ± 0.22	2.95 ± 0.44	2.98 ± 0.29
0.05% BAC	1.81 ± 0.04	2.00 ± 0.09	1.74 ± 0.02	1.93 ± 0.10	1.78 ± 0.10	2.04 ± 0.06	1.80 ± 0.07	2.12 ± 0.06	1.78 ± 0.02	2.00 ± 0.06	1.66 ± 0.14	1.82 ± 0.08	1.86 ± 0.06	1.94 ± 0.05	1.84 ± 0.04	2.03 ± 0.16
0.2% BAC	3.10 ± 0.04	3.32 ± 0.09	3.02 ± 0.06	3.27 ± 0.10	3.26 ± 0.08	>4.00	3.12 ± 0.06	>4.00	3.09 ± 0.10	>4.00	2.73 ± 0.22	>4.00	2.98 ± 0.38	>4.00	3.16 ± 0.16	>4.00

*The log reduction value was calculated to evaluate disinfectant effectiveness under each condition and was expressed as mean ± standard deviation. Additionally, the log reduction value of the condition wherein the virus was inactivated below the measurement limit (10^1 FFUs) was 4 or more and was expressed as >4.00. BAC, benzalkonium chloride; CHG, chlorhexidine gluconate; EA, ethyl alcohol; H5N1-Ky, A/crow/Kyoto/53/04 (H5N1); H5N1-Eg, A/chicken/Egypt/CL6/07 (H5N1); H7N9, A/Anhui/1/23 (H7N9); H5N3, A/Duck/Hong Kong/820/80 (H5N3); H5N9, A/Turkey/Ontario/7732/66 (H5N9); H3N2, Clinical strain (H3N2); H1N1-PR8, A/Puerto Rico /8/1934 (H1N1); H1N1-Ok-pdm, A/Osaka/64/2009 (H1N1); IPA, isopropanol.

Appendix Table 3. Evaluating the effectiveness of disinfectants against various recombinant influenza viruses*

	Log reduction, mean + standard deviation											
	rH5N1-H5N3-NA		rH5N1-H5N3-HA		rH5N3-H5N1-NA		rH5N3-H5N1-NS		rH5N3-H5N1-M		rH5N3-H5N1-HA	
Disinfectant	in vitro	on skin	in vitro	on skin	in vitro	on skin	in vitro	on skin	in vitro	on skin	in vitro	on skin
20% EA	0.09 + 0.03	1.11 + 0.03	0.09 + 0.04	1.06 + 0.03	0.07 + 0.08	1.09 + 0.06	0.15 + 0.03	1.11 + 0.07	0.05 + 0.03	1.09 + 0.06	0.06 + 0.07	1.05 + 0.10
32% EA	0.72 + 0.04	2.07 + 0.01	0.64 + 0.03	1.97 + 0.14	0.19 + 0.04	1.04 + 0.04	0.72 + 0.03	2.10 + 0.05	0.67 + 0.04	1.89 + 0.21	0.69 + 0.10	1.88 + 0.09
34% EA	1.73 + 0.06	2.40 + 0.16	1.85 + 0.10	2.24 + 0.20	0.69 + 0.08	1.26 + 0.06	1.89 + 0.04	2.59 + 0.04	1.54 + 0.09	2.36 + 0.07	1.41 + 0.10	2.22 + 0.15
36% EA	>4.00	>4.00	>4.00	>4.00	1.75 + 0.09	1.63 + 0.10	>4.00	>4.00	>4.00	>4.00	>4.00	>4.00
40% EA	>4.00	>4.00	>4.00	>4.00	>4.00	>4.00	>4.00	>4.00	>4.00	>4.00	>4.00	>4.00

*The reaction time with the disinfectant was 15 seconds. The log reduction value was calculated to evaluate disinfectant effectiveness under each condition and was expressed as mean + standard deviation. Additionally, the log reduction value of the condition wherein the virus was inactivated below the measurement limit (in vitro: 10^{1} FFUs, *on skin*: $10^{1.6}$ FFUs) was 4 or more and was expressed as ">4.00." EA; Ethyl alcohol, IPA; Isopropanol, CHG; Chlorhexidine gluconate, BAC; Benzalkonium chloride, A/crow/Kyoto/53/04 (H5N1) was recombined with the NA or HA gene of A/Duck/Hong Kong/820/80 (H5N3), and the recombinant viruses were designated as rH5N1-H5N3-NA and rH5N1-H5N3-HA, respectively. In addition, A/Duck/Hong Kong/820/80 (H5N3) was recombined with the NA, NS, M, or HA gene of A/crow/Kyoto/53/04 (H5N1), and the recombinant viruses were designated as rH5N3-H5N1-NA, rH5N3-H5N1-NS, rH5N3-H5N1-M, or rH5N3-H5N1-HA, respectively. EA, ethyl alcohol.

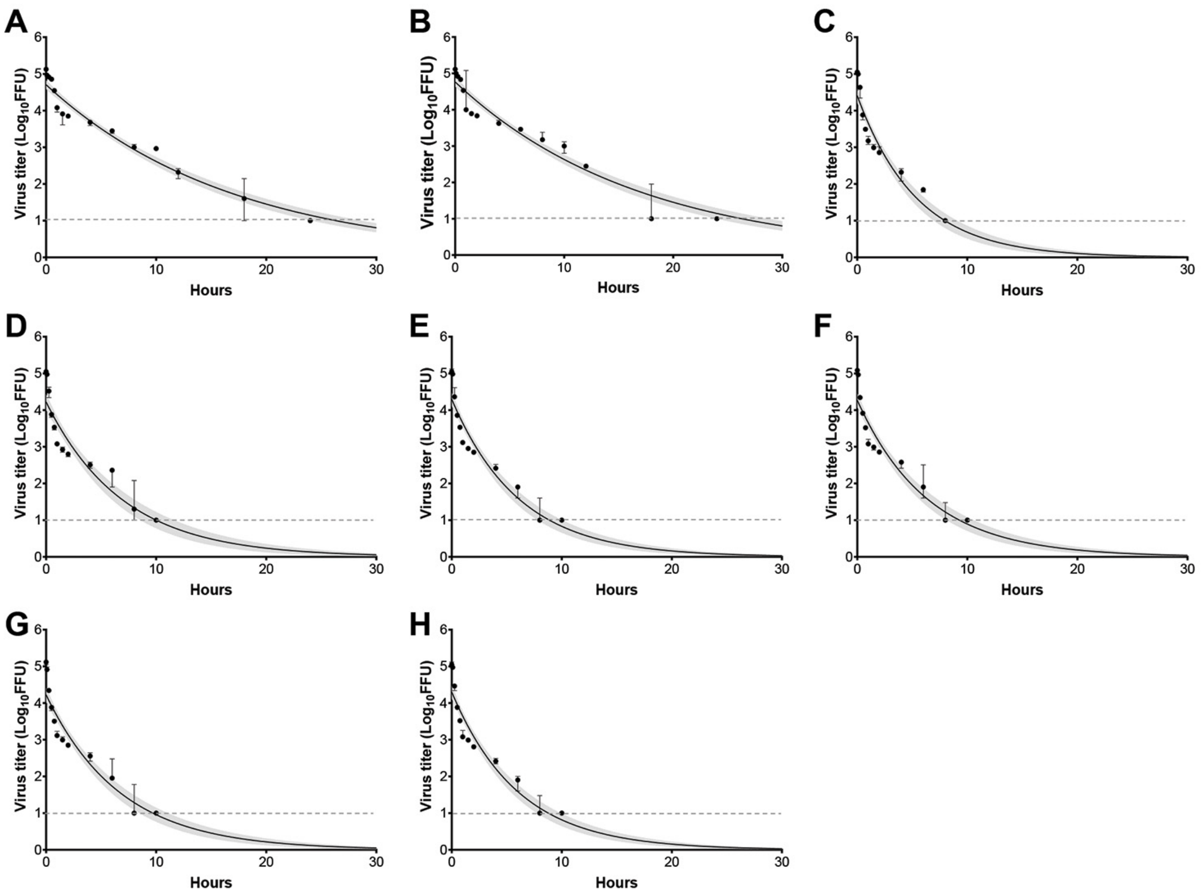

Appendix Figure 1. Stability of various subtypes of influenza viruses on a plastic surface. Stability data are shown for A) H5N1-Ky, A/crow/Kyoto/53/04 (H5N1); B) H5N1-Eg, A/chicken/Egypt/CL6/07 (H5N1); C) H7N9, A/Anhui/1/23 (H7N9); D) H5N3, A/Duck/Hong Kong/820/80 (H5N3); E) H5N9, A/Turkey/Ontario/7732/66 (H5N9); F) H3N2, a clinical H3N2 strain; G) H1N1-PR8, A/Puerto Rico/8/1934 (H1N1); and H) H1N1-Ok-pdm, A/Osaka/64/2009 (H1N1). The elapsed time was defined as an explanatory variable (x-axis), and the log of the virus titer was defined as an explained variable (y-axis). Least-squares linear-regression analysis was performed with a logarithmic link function for each virus to generate a regression curve. The upper and lower confidence limits are represented by dotted curves. The data shown are expressed as median ± standard error of the mean for 3 independent experiments.

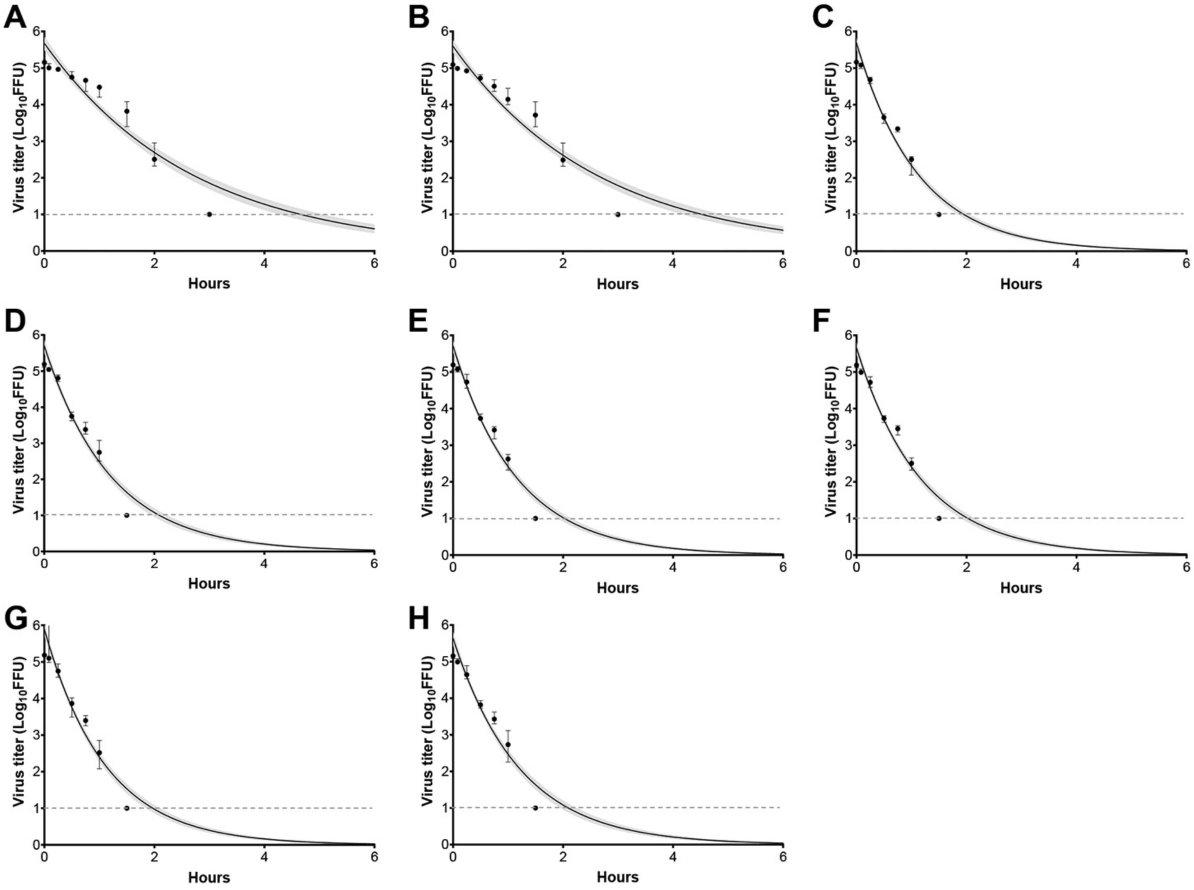

Appendix Figure 2. Stability of various subtypes of influenza viruses on the human skin surface. Stability data are shown for A) H5N1-Ky, A/crow/Kyoto/53/04 (H5N1); B) H5N1-Eg, A/chicken/Egypt/CL6/07 (H5N1); C) H7N9, A/Anhui/1/23 (H7N9); D) H5N3, A/Duck/Hong Kong/820/80 (H5N3); E) H5N9, A/Turkey/Ontario/7732/66 (H5N9); F) H3N2, a clinical H3N2 strain; G) H1N1-PR8, A/Puerto Rico/8/1934 (H1N1); and H) H1N1-Ok-pdm, A/Osaka/64/2009 (H1N1). The elapsed time was defined as an explanatory variable (x-axis), and the log virus titer was defined as an explained variable (y-axis). Least-squares linear-regression analysis was performed with a logarithmic link function for each virus to generate a regression curve. The upper and lower confidence limits are represented by dotted curves. The data shown are expressed as median + standard error of the mean for 3 independent experiments.

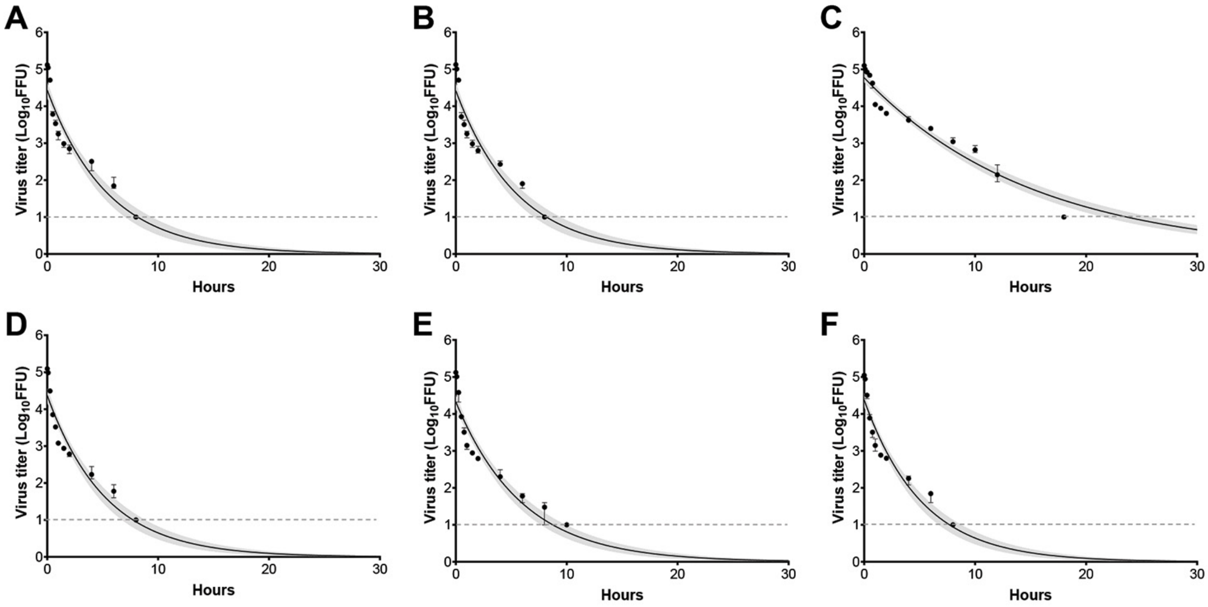

Appendix Figure 3. Stability of various recombinant viruses on a plastic surface. A/crow/Kyoto/53/04 (H5N1) was recombined with the *NA* or *HA* gene of A/Duck/Hong Kong/820/80 (H5N3), and the recombinant viruses were designated as A) rH5N1-H5N3-NA or B) rH5N1-H5N3-HA. In addition, A/Duck/Hong Kong/820/80 (H5N3) was recombined with the *NA*, *NS*, *M*, or *HA* gene of A/crow/Kyoto/53/04 (H5N1), and the recombinant viruses were designated as C) rH5N3-H5N1-NA, D) rH5N3-H5N1-NS, E) rH5N3-H5N1-M, or F) rH5N3-H5N1-HA. The elapsed time was defined as an explanatory variable (x-axis), and the log virus titer was defined as an explained variable (y-axis). Least-squares linear-regression analysis was performed with a logarithmic link function for each virus to generate a regression curve. The upper and lower confidence limits are represented by dotted curves. The data shown are expressed as median $\pm$ standard error of the mean for 3 independent experiments.

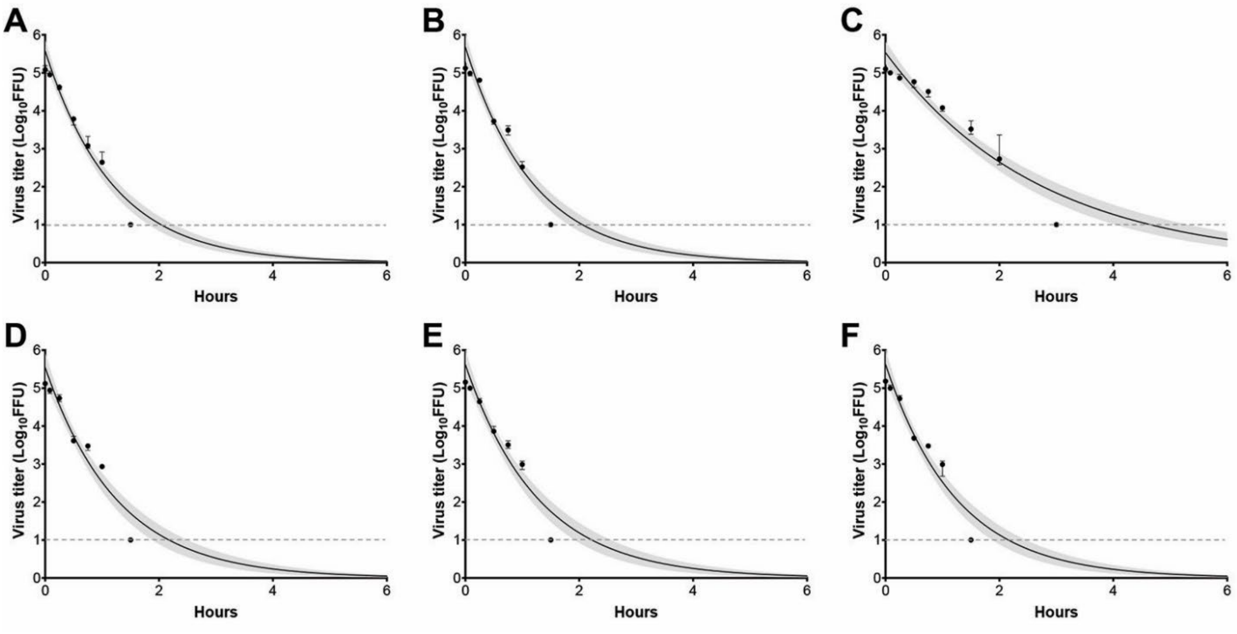

Appendix Figure 4. Stability of various recombinant viruses on the surface of human skin. A/crow/Kyoto/53/04 (H5N1) was recombined with the *NA* or *HA* gene of A/Duck/Hong Kong/820/80 (H5N3), and the recombinant viruses were designated as A) rH5N1-H5N3-NA or B) rH5N1-H5N3-HA. In addition, A/Duck/Hong Kong/820/80 (H5N3) was recombined with the *NA*, *NS*, *M*, or *HA* gene of A/crow/Kyoto/53/04 (H5N1), and the recombinant viruses were designated as C) rH5N3-H5N1-NA, D) rH5N3-H5N1-NS, E) rH5N3-H5N1-M, or F) rH5N3-H5N1-HA. The elapsed time was defined as an explanatory variable (x-axis), and the log virus titer was defined as an explained variable (y-axis). Least-squares linear-regression analysis was performed with a logarithmic link function for each virus to generate a regression curve. The upper and lower confidence limits are represented by dotted curves. The data shown are expressed as median + standard error of the mean for >3 independent experiments.
